# Complete Genome Sequence of Micrococcus luteus Strain SGAir0127, Isolated from Indoor Air Samples from Singapore

**DOI:** 10.1128/MRA.00646-19

**Published:** 2019-10-10

**Authors:** Shruti Ketan Kutmutia, Daniela I. Drautz-Moses, Akira Uchida, Rikky W. Purbojati, Anthony Wong, Kavita K. Kushwaha, Alexander Putra, Balakrishnan N. V. Premkrishnan, Cassie E. Heinle, Vineeth Kodengil Vettath, Ana Carolina M. Junqueira, Stephan C. Schuster

**Affiliations:** a Singapore Centre for Environmental Life Sciences Engineering, Nanyang Technological University, Singapore; b Departamento de Genética, Instituto de Biologia, Universidade Federal do Rio de Janeiro, Rio de Janeiro, Brazil; University of California, Riverside

## Abstract

Micrococcus luteus strain SGAir0127 was isolated from indoor air samples collected in Singapore. The assembly, based on single-molecule real-time sequencing reads, resulted in two contigs, one chromosomal contig with a length of 2.57 Mbp and one nonchromosomal contig of 8.68 kbp. The genome has a total of 2,564 genes.

## ANNOUNCEMENT

Micrococcus luteus, previously known as Micrococcus lysodeikticus, was first identified by Fleming (1) while discovering lysozyme. It is a Gram-positive, coccus-shaped, aerobic bacterium arranged in clusters or tetrads belonging to the actinomycete branch of taxonomy. It survives and grows in a wide variety of environments, including oligotrophic environments (2), owing to its ability to biodegrade atypical substrates like carbofuran, naphthalene, environmental pesticides (3), and itaconate (4), as well as common substrates. Micrococcus luteus is also known to be opportunistically pathogenic (5) and putrefactive (6).

Micrococcus luteus strain SGAir0127 was isolated from indoor air samples collected in Singapore (global positioning system [GPS] coordinates 1.3451N, 103.6789E) using a single-stage Andersen impactor (SKC, USA) to impact air onto a Trypticase soy agar (TSA) plate (Becton, Dickinson, USA). After the initial incubation at 30°C, a pure culture of strain SGAir0127 was achieved by repeated restreaking on fresh TSA plates. A colony was then inoculated in lysogeny broth (LB) (Merck, USA) at 30°C overnight and used for DNA extraction. Genomic DNA was extracted using the Wizard Genomic DNA purification kit (Promega, USA), following the manufacturer’s protocol. Library preparation was performed using the SMRTbell template prep kit 1.0 (Pacific Biosciences, USA), followed by single-molecule real-time (SMRT) sequencing on the Pacific Biosciences RS II platform.

The Hierarchical Genome Assembly Process (HGAP) version 3 (with default values) (7) from PacBio’s SMRT Analysis 2.3.0 package assembled a total of 60,672 reads into two contigs. After Quiver polishing (with default values) (7), a chromosomal contig with a length of 2,570,740 bp and 201-fold coverage and a nonchromosomal contig with a length of 86,829 bp and 207-fold coverage were found. A positive GC skew of 72.9% was observed in the chromosomal contig, which is characteristic of actinomycetes (8).

Phyla-AMPHORA was run using MarkerScanner.pl with added -DNA flag and MarkerAlignTrim.pl and with options -WithReference and -OutputFormat phylip, followed by Phylotyping.pl, with default parameters (9). It was used for taxonomic identification and showed that the assembled genome has 99.5% marker identity with Micrococcus luteus. Microbial Species Identifier (MiSI) (10), based on the genome-wide average nucleotide identity (ANI) metric, was run against a database of 6,387 bacterial refseq genomes using ANICalculator with default parameters, a text filter for “type, synonym type, proxytype,” and a subsequent getorf -find 3 option. The results showed 97.8% identity to Micrococcus luteus NCTC 2665 with an alignment fraction of 0.60.

PROKKA version 1.12 (added parameters –force –compliant –addmrna –rfam) was used for gene annotation and predicted 2,495 protein-coding genes (11). The genome contained 6 rRNAs, 52 tRNAs, 1 transfer-messenger RNA (tmRNA), and 10 noncoding RNAs (ncRNAs), for a total of 69 RNA genes. In addition, a CRISPR element with 31 repeat units in the chromosomal contig and a CRISPR sequence with 6 repeat units in the nonchromosomal contig were also observed.

Rapid Annotations using Subsystems Technology (RAST) v2 (12–14), run with default parameters with an additional frameshift fix and ClassicRAST annotation scheme, showed the potential ability of this bacterium to metabolize a wide range of organic compounds, such as isoprenoids, aromatics, and carbohydrates, as well as inorganic ions/compounds. RAST identified 68 genes for stress response, including carbon starvation and oxidative stress as a survival strategy (15), as these are the obstacles most likely to be faced by bacteria in the air. Dormancy, persistence, and resuscitation genes were also annotated, but a reduced number was observed compared to other actinomycetes (16). Furthermore, the presence of fluoroquinolone resistance genes is an intriguing feature of this strain.

### Data availability.

The complete genome sequence of Micrococcus luteus strain SGAir0127 has been deposited in DDBJ/EMBL/GenBank under accession number CP025616, and the associated nonchromosomal contig was deposited under accession number CP025617. The Sequence Read Archive files are available under accession number SRR8948643.
